# Influence of human chorionic gonadotrophin during ovarian stimulation: an overview

**DOI:** 10.1186/s12958-020-00639-3

**Published:** 2020-08-06

**Authors:** Johan Smitz, Peter Platteau

**Affiliations:** 1 Follicle Biology Laboratory, Vrije Universiteit Brussel, Laarbeeklaan, 103, 1090 Brussels, Belgium; 2 Centre for Reproductive Medicine, Universitair Ziekenhuis Brussel, Vrije Universiteit Brussel, Brussels, Belgium

**Keywords:** Luteinising hormone, Human chorionic gonadotrophin, Human menopausal gonadotrophin, Assisted reproductive technology, Ovarian stimulation

## Abstract

It is widely known that luteinising hormone (LH) and human chorionic gonadotrophin (hCG) are integral in the female reproductive lifecycle. Due to the common binding site and similarity in molecular structure, they were previously thought to have overlapping roles. However, with the development of both purified urinary-derived and recombinant gonadotrophins, the individual characteristics of these molecules have begun to be defined. There is evidence to suggest that LH and hCG preferentially activate different signalling cascades and display different receptor-binding kinetics. The data generated on the two molecules have led to an improved understanding of their distinct physiological functions, resulting in a debate among clinicians regarding the most beneficial use of LH- and hCG-containing products for ovarian stimulation (OS) in assisted reproductive technologies (ARTs). Over the past few decades, a number of trials have generated data supporting the use of hCG for OS in ART. Indeed, the data indicated that hCG plays an important role in folliculogenesis, leads to improved endometrial receptivity and is associated with a higher quality of embryos, while presenting a favourable safety profile. These observations support the increased use of hCG as a method to provide LH bioactivity during OS. This review summarises the molecular and functional differences between hCG and LH, and provides an overview of the clinical trial data surrounding the use of products for OS that contain LH bioactivity, examining their individual effect on outcomes such as endometrial receptivity, oocyte yield and embryo quality, as well as key pregnancy outcomes.

## Background

Human chorionic gonadotrophin (hCG) and luteinising hormone (LH) are two hormones of the female reproductive system that, despite their similar structures, common receptor and overlapping physiological roles, have distinct differences in terms of their bioactivity and physiological function [1, 2]. Follicular growth and development are conventionally associated with LH, which enhances ovarian steroidogenesis through the ‘two-cell, two-gonadotrophin’ theory [3–5]. In contrast, hCG has mostly been identified as the ‘pregnancy hormone’ due to its role in embryo implantation and pregnancy maintenance [6, 7]. However, hCG expression has also been confirmed in non-pregnant women of reproductive age, where it is thought to play a role during the normal menstrual cycle, as well as in men and post-menopausal women [2, 8]. In addition, a number of trials carried out over the past decade have described data supporting the use of hCG for ovarian stimulation (OS) in assisted reproductive technologies (ARTs), suggesting a role in folliculogenesis [2, 8–12]. *Post-hoc* analyses of these data suggest improved endometrial receptivity and embryo quality during OS cycles with hCG-containing products versus those without [11–14]. These observations have added strength to the increased use of hCG to provide LH activity during OS in ART [11, 12]. However, an increased understanding of the key differences between LH and hCG has revealed significant variance in their physiological functions, resulting in a debate among clinicians regarding the most beneficial use of LH- and hCG-containing products for OS [1].

This review will discuss the differences between hCG and LH at the molecular and functional levels, explore the evolution of their pharmacological use in ART, before examining the clinical trial data surrounding the use of products that contain LH bioactivity derived from LH or hCG in OS.

## Biological differences between hCG and LH

### Molecular structure

**Fig. 1:**
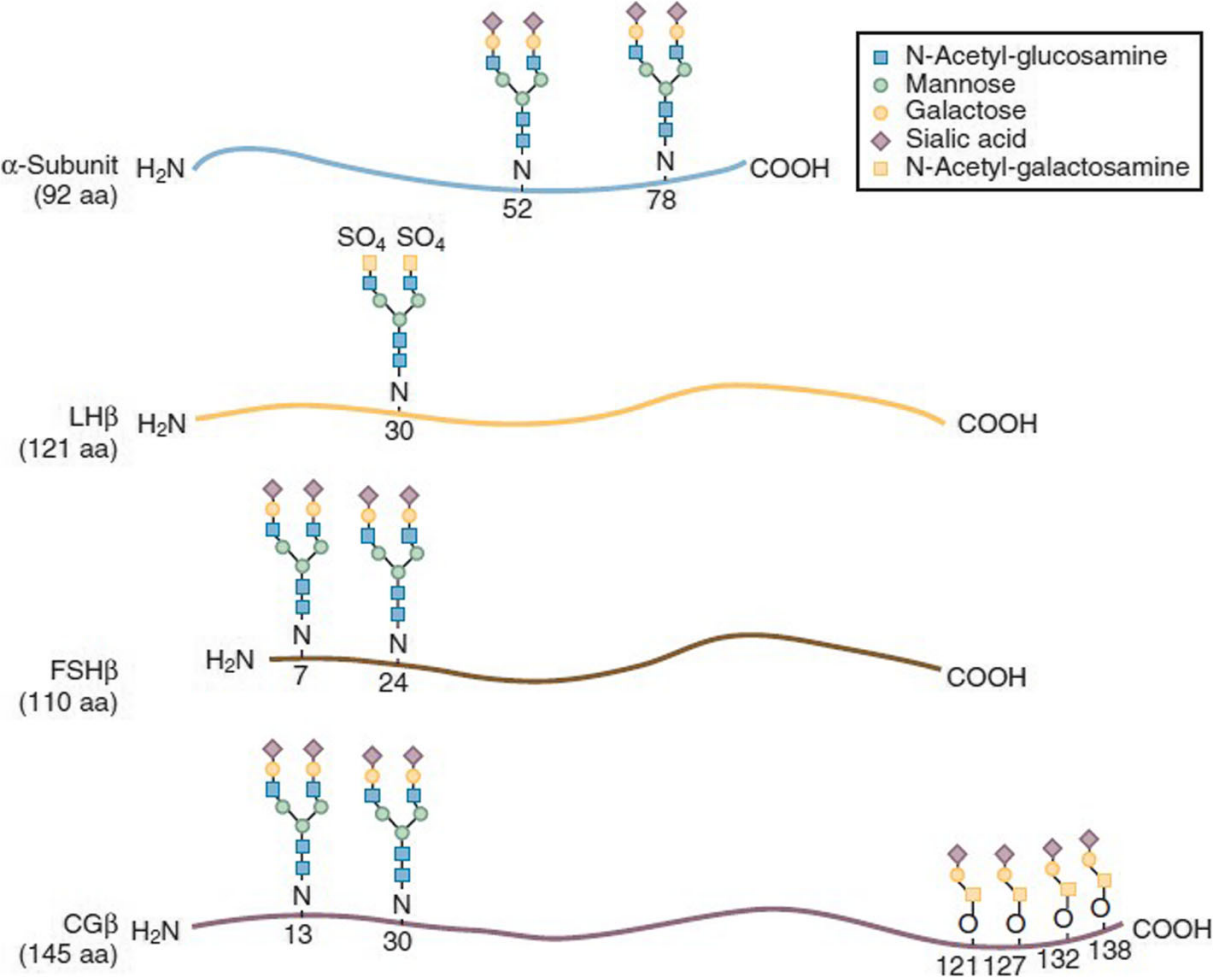
Structural similarities and differences between FSH, hCG and LH [15]. CG, chorionic gonadotrophin; COOH, carboxylic acid; FSH, follicle-stimulating hormone; hCG, human chorionic gonadotrophin; LH, luteinising hormone

hCG and LH are structurally similar molecules; they both belong to a family of heterodimeric glycoprotein hormones [1, 6] and consist of highly glycosylated, non-covalently linked alfa and beta subunits (Fig. 1). The alfa subunit, consisting of 92 amino acids, is common not only in hCG and LH but also in FSH and thyroid-stimulating hormone [1, 6, 16]. The beta subunits differ in length and confer structural individuality, as well as specificity of physiological activity to each hormone. The main difference between beta subunits of hCG and LH lies with the 24-amino-acid carboxy-terminal peptide (CTP) extension sequence, which contains four O-linked carbohydrate side chains (Fig. 1) [17, 18]. The LH precursor undergoes cleavage of the CTP sequence during protein synthesis to become a protein that is 121 amino acids in length [1, 17, 18]. In addition, the beta subunits of hCG and LH have differing levels of post-translational glycosylation, which has an important impact on the structural conformation of the molecule, as well as its bioactivity [1, 19–21].

Further differences between hCG and LH are displayed in vivo through the existence of multiple isoforms of each molecule. Owing to variation in the content of terminal sialic acid, LH and hCG display extensive charge heterogeneity depending on the isoform. The isoforms of LH have an isoelectric point (pI) ranging from < 4.0 to > 7.2, whereas the pI of hCG ranges from 3 to 7 due to the higher acidity of some hCG isoforms [22, 23]. The composition of LH isoforms appears to vary throughout the reproductive lifecycle, with younger women presenting isoforms with a shorter half-life and decreased potency. The different hCG isoforms have been previously reviewed in detail [1, 8, 18, 24–28]. The individual isoforms of hCG and LH exhibit different biological functions, which suggests that there may be a unique role for each isoform that could have functional significance [1].

### Physiological role in vivo

The unique presence of the CTP extension confers hCG a markedly increased half-life in comparison to LH (approximately 24–34 h vs approximately 30–60 min, respectively [29, 30]). The specific effect of CTP has previously been demonstrated in pharmacokinetics studies investigating the addition of the CTP extension to the wild-type recombinant FSH beta (rFSH beta) molecule. FSH is commonly used in ART protocols for OS, but due to the relatively rapid clearance of the wild-type molecule, daily administration is required. Investigators aimed to extend the clearance of rFSH beta in order to reduce the number of injections required during ART procedures. To achieve this, a chimeric rFSH-CTP was created by fusing wild-type rFSH beta with a CTP that has an identical amino-acid sequence to the CTP found on hCG [31]. The resulting rFSH-CTP was then tested in pituitary-suppressed females, resulting in a mean elimination half-life of between 60 and 75 h, which is approximately double that of rFSH beta [32, 33]. When relating these study outcomes to hCG and LH, as both molecules have a common structure apart from the CTP extension, it can be reasoned that hCG is simply a longer-acting version of LH and could be thought of as “LH-CTP”.

**Fig. 2:**
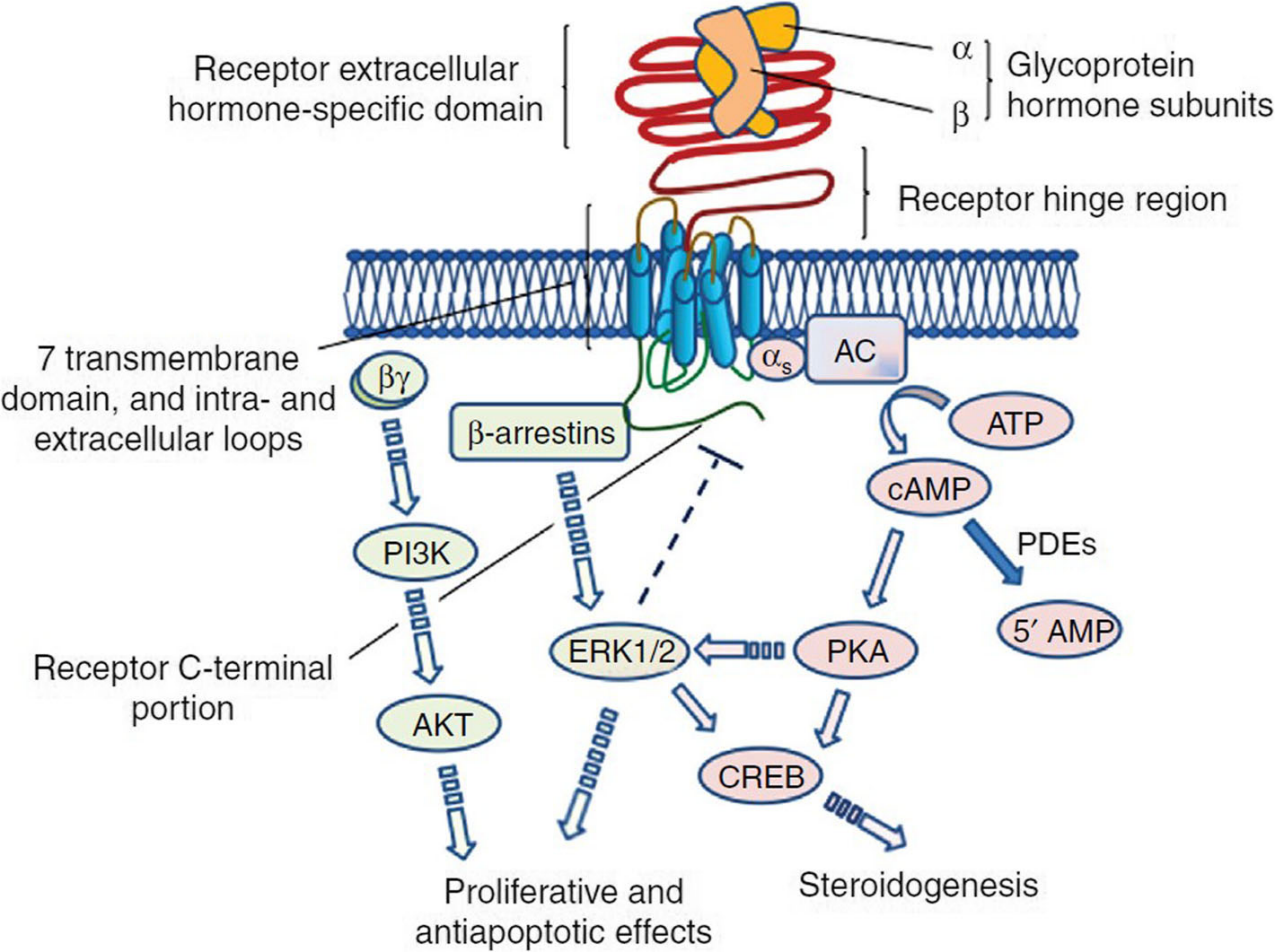
hCG- and LH-induced signalling cascades via LH/CGR [19]. AC, adenylyl cyclase enzyme; AKT, protein kinase B; ATP, adenosine tri-phosphate; cAMP, cyclic adenosine monophosphate; CREB, cAMP response element-binding protein; ERK1/2, extracellular regulated kinases 1 and 2; hCG, human chorionic gonadotrophin; LH, luteinising hormone; LH/CGR, luteinising hormone/choriogonadotrophin receptor; PDE, phosphodiesterase enzyme; Pl3K, phosphoinositide 3-kinases; PKA, protein kinase A

The CTP extension also confers other physiological differences between hCG and LH, in addition to their half-lives. The bioactivities of endogenous hCG and LH are mediated through binding to a common receptor and triggering of specific signalling cascades (Fig. 2) [34, 35]. hCG and LH both bind to the LH/choriogonadotrophin receptor (LH/CGR), a G-protein-coupled receptor consisting of a large, extracellular ligand-binding domain connected to a transmembrane domain via a hinge region [6, 19, 36]. The extracellular domain also has a number of leucine-rich repeats, to which hCG and LH bind in distinct regions with strong affinity and high specificity [19, 36]. The LH/CGR differentiates between LH and hCG binding through its hinge region, which mediates structural and spatial rearrangements in the receptor by transmitting the ligand-induced extracellular conformational change to the transmembrane region, triggering the activation of complex intracellular pathways [19, 30, 34]. Receptor-activated intracellular pathways include the cyclic adenosine monophosphate (cAMP)/protein kinase A pathway, which results in steroidogenesis and increased progesterone synthesis, and the β-arrestin and extracellular signal-regulated kinase 1/2 (ERK1/2)/protein kinase B (AKT) pathway, which induces proliferative and anti-apoptotic pathways [30]. However, the relative potency of the activation of each pathway is dependent on the ligand [30, 37].

A number of in vitro studies have demonstrated the differences in intracellular responses between LH and hCG via LH/CGR. These studies indicate that LH binding results in a more potent activation of the proliferative and anti-apoptotic ERK1/2 and AKT pathways, whereas hCG has a higher potency for activation of the steroidogenic cAMP pathways [37–39]. In addition, there is evidence to suggest that steroid hormone production differs between hCG and LH. Whereas LH and hCG both fully promote testosterone production, LH only partially stimulates progesterone production, with approximately half the potency of hCG [39].

In keeping with the influence of the CTP component on the half-life of hCG, an in vitro gonadotrophin kinetic binding study using rat LH/CGR revealed that, although hCG and LH have a similar association rate to the receptor (3.4 × 10^8^ and 4.0 × 10^8^ M^− 1^ min^− 1^ for hCG and human luteinising hormone [hLH], respectively), hCG displays a markedly longer dissociation rate in contrast to LH (25 h vs 9.2 h, respectively, measured via the half-times of the bound ligands) [40]. This indicates that hCG interacts with the rat LH/CGR for an increased duration of time; whether these findings translate to hCG displaying a higher binding affinity to the LH/CGR in humans is still to be determined. A recent study investigating the reversibility of hCG or hLH stimulation of mouse Leydig tumour cells found hLH to have a faster rate of dissociation (approximately 9 h vs 25 h) from the LH receptor in comparison to hCG [34, 41]. In addition, a separate study has shown that recombinant hCG (rhCG) activation of the receptor induces faster cAMP accumulation in comparison to recombinant human LH (rhLH) (68.82 ± 22.30 pM vs 459.49 ± 105.35 pM), suggesting different kinetics with the receptor between the two hormones [34]. Overall, preclinical data suggest that hCG dissociates from the receptor more slowly than LH, which leads to an increased duration of action of approximately 16 h displayed at the LH/CGR and an increased potency for intracellular signalling activation [34]. Further studies are required to confirm the biological activity of the differential binding affinities of each hormone [30, 34].

### Molecular evaluation of LH or hCG action on follicle cells in vivo

Over the last decade, the primary role of LH bioactivity in oocyte competence has been clarified from studies in animal models (mouse and bovine) [42–45] and human ART experience [46]. These studies indicated that the epidermal growth factor (EGF) network is essential for LH signal transduction in the follicle and have provided insights into how this signalling network coordinates nuclear and cytoplasmic maturation in the oocyte before ovulation. In mural granulosa, mobilisation and release of the three EGF-like factors, amphiregulin, epiregulin and betacellulin at ovulation are dependent on an increasingly sustained tonus of LH in the last days before ovulation [42, 43]. The LH tonus in the days before ovulation will also determine the steroid production in the follicle (see “Function in Reproduction” section for further details), which influences the subsequent implantation capacity in a natural cycle [44–46]. Hence, LH bioactivity will affect the ripening of the follicle and determine oocyte potential for implantation.

As illustrated throughout this review, LH bioactivity during ART treatment can be heavily influenced by the stimulation regimen chosen (type of gonadotrophin-releasing hormone (GnRH) co-treatment, type of gonadotrophin preparation, and the type of ovulation trigger applied). Based on early studies using rLH as an ovulatory stimulus, it is clear that prolonged LH bioactivity would be needed to prevent shortening of the luteal phase and support corpus luteum function and endometrial preparation [47]. Several reports have pointed to major differences at the gene-expression and protein level in granulosa and cumulus cells obtained at in vitro fertilisation (IVF) pick-up depending on: (i) whether the stimulation regimen included LH bioactivity (rFSH alone vs supplemental LH bioactivity) [48, 49] and (ii) the compounds used for ovulation triggering (hCG trigger vs GnRH agonist trigger) [50, 51]. Although the use of a GnRH agonist trigger in an antagonist stimulation treatment has firmly reduced the risks of hyperstimulation, it is not always as effective as the conventional hCG trigger in terms of maturation of oocytes and supporting the luteal phase. When a GnRH agonist trigger is administered, the response is uniquely driven by LH and FSH, which are mobilised depending on pituitary reserve availability. Whereas, injection of an hCG trigger delivers ‘long acting’ LH bioactivity, without increasing FSH level [52]. It has been suggested that a double trigger (an agonist trigger concomitant with an hCG dose) might induce a more effective stimulation trigger via the additional pituitary mobilisation of FSH. Based on the gene signatures obtained from granulosa cells, double triggering may lead to improved oocyte and embryo quality compared with hCG triggering alone [53, 54].

### Function in reproduction

The diverse activities exhibited by hCG and LH at the LH/CGR, and the different intracellular signalling pathways activated by these hormones, are all indicators of the distinct functions that each hormone plays in reproduction [18, 55]. Although it is widely accepted that LH primarily acts to regulate follicular growth, development and maturation, recent evidence suggests that hCG may also have an important role throughout the menstrual cycle and in menopausal women [8, 27, 56, 57].

LH is produced at a consistent level during the early follicular phase of a normal menstrual cycle, stimulating steroidogenesis and the conversion of pregnenolone to androgens within ovarian follicular theca cells. Follicular granulosa cells undergo simultaneous stimulation by FSH, resulting in the production of the enzyme aromatase, which converts androgens produced in the theca cells to oestrogens [2, 58]. This is known as the ‘two-cell, two-gonadotrophin’ theory of follicular development, and it is thought that the resultant increase in oestradiol levels within the follicular microenvironment may assist in the selection of a dominant follicle [59]. A surge in LH levels at the midpoint in the menstrual cycle, induced via a positive feedback mechanism due to rising oestradiol secreted by the preovulatory follicle, triggers ovulation of the mature follicle. The LH surge also stimulates the initial formation of the corpus luteum, luteinisation of the granulosa cells and early progesterone synthesis [59].

The traditional view is that, after implantation, hCG produced by the trophoblast cells is believed to take over the control of progesterone production by granulosa and corpus luteal cells from LH [29]. Progesterone is vital to promote and maintain pregnancy at this early stage as it primes the endometrium for implantation of the forthcoming embryo and prevents menstrual bleeding, continuing to support the endometrial organisation for 3–4 weeks following a successful implantation. Although hCG only promotes progesterone production for the first 3–4 weeks, the hCG concentration continues to rise, reaching its peak at 10 weeks’ gestation [8, 29]. At this point, the placenta takes over the production of hCG, with levels gradually reducing over the remaining weeks [60–62]. The presence of hCG throughout the entire duration of pregnancy, however, indicates a role beyond progesterone production [29]. Additional known functions of hCG include: promoting angiogenesis and vasculogenesis of the uterine vasculature to increase the blood supply to the placenta and developing foetus; promoting the fusion and differentiation of placental cytotrophoblasts into syncytiotrophoblasts (which, in turn, produce more hCG); promoting growth and differentiation of foetal organs; promoting umbilical cord growth; and development and inhibition of macrophage function to prevent rejection of the foetal and placental tissue [8, 18, 29, 61, 63, 64].

Pituitary hCG secretions can be seen to mimic the pulsatile pattern of LH expression throughout the normal menstrual cycle, with hCG peaking in parallel with the LH surge, albeit on average at only 3% of the concentration of LH [2, 8, 65, 66]. It has been suggested that the longer circulatory half-life of hCG may increase the peak range of LH activity, with evidence from 185 women indicating that up to one-third of the LH activity during the follicular phase may be derived from hCG [2, 8, 9]. Together, these data suggest that hCG function may overlap with that of LH throughout the menstrual cycle and support LH in the stimulation of follicular maturation and induction of ovulation, as well as supporting a rise in progesterone levels during the early luteal phase [8, 18]. There is, nevertheless, also a possibility that hCG production is incidental, occurring as a result of GnRH-mediated LH and FSH secretion from the pituitary [8].

A study by Snyder et al. (2005) provided data indicating that hCG levels increase with age, and concentrations of > 5 IU/L in post-menopausal women should be considered to be normal [57]. Considering that standard tests use a cut-off concentration of > 5 IU/L hCG for a positive pregnancy, this is of clinical importance as women of menopausal age are at risk of receiving false-positive results [57]. This also provides further evidence for a role of hCG outside of pregnancy.

Owing to the similarities in their molecular structures and data indicating that hCG has a role throughout the menstrual cycle, LH and hCG are currently used interchangeably in ART protocols to drive OS [1, 67].

### Evolution of the pharmacological use of hCG and LH in ART

The prospect of developing gonadotrophin preparations for clinical use was first explored over a century ago; researchers hoped to harness the physiological actions of the naturally occurring hormones for clinical use to induce OS in infertile women in an attempt to achieve a successful pregnancy. Since then, a number of different gonadotrophin preparations have been produced and successfully used for OS in ART (Table 1). The first hCG preparation was extracted from human placental tissue and made commercially available in 1931. Initial investigations by Ascheim and Zondek in 1927 revealed that hCG in the blood and urine of pregnant women was able to induce follicular maturation and ovulation when injected into female mice, and subsequent investigations showed hCG production to be localised to the placenta [73, 74]. Clinical use of this preparation showed that, when administered alone, hCG was unable to promote either follicular development or ovulation, hence indicating that FSH stimulation is vital during the follicular phase [73].

**Table 1:** Gonadotrophin products used in ovarian stimulation [21, 68–72]

Brand name	Urinary/recombinant	Active ingredients
Menopur® Merional®	Urinary	Highly purified human menopausal gonadotrophin (FSH + hCG)
Humegon® Menogon® Pergonal® Repronex®	Urinary	Human menopausal gonadotrophin (FSH + LH)
Fostimon® Bravelle® Metrodin® Fertinex® Fertinorm®	Urinary	Urofollitropin (highly purified FSH)
Choragon® Pregnyl® Novarel® Profasi® Predalon® Gonasi® Brevactid® Biogonadyl® Primogonyl® Endocorion® Corion®	Urinary	Human chorionic gonadotrophin (hCG)
Gonal-F®	Recombinant	Follitropin alfa (rFSH)
Puregon® Follistim®	Recombinant	Follitropin beta (rFSH)
Elonva®	Recombinant	Corifollitropin alfa (long-acting rFSH)
Pergoveris®	Recombinant	Follitropin alfa and lutropin alfa (rFSH and rLH)
Luveris®	Recombinant	Lutropin alfa (rLH)
Ovitrelle® Ovidrelle® Ovidrel®	Recombinant	Recombinant human chorionic gonadotrophin (rhCG)

*FSH* follicle-stimulating hormone, *hCG* human chorionic gonadotrophin, *LH* luteinising hormone, *rFSH* recombinant follicle-stimulating hormone, *rhCG* recombinant human chorionic gonadotrophin, *rLH* recombinant luteinising hormone

Gonadotrophins extracted from the urine of postmenopausal women, known as human menopausal gonadotrophin (hMG), have traditionally been used to stimulate folliculogenesis in ART. The first purified preparations from urine contained a mixture of FSH, hCG and LH in varying amounts, sometimes with as little as 5% purity [73–75]. Improvements in purification techniques enabled the development of standardised batches of hMG containing 75 IU each of LH and FSH activity, as well as the individual development of urinary FSH or urinary LH preparations [21, 73]. Highly purified FSH (HP-FSH) became widely available in 1995 and contained < 0.1 IU of LH activity [76]. It was extracted to yield an increase in purity from 1 to 95% (containing only 5% biological contamination), which has allowed for a reduction in the amount of injected protein so that it could be delivered subcutaneously at a much smaller dose than the original purified FSH [73, 74]. Commercially available, highly purified hMG (HP-hMG) was subsequently developed, containing a 1:1 ratio of FSH (75 IU) and LH bioactivity (75 IU) that was predominantly derived from hCG [77]. In parallel to the evolution of urinary-derived gonadotrophins, recombinant gonadotrophins were developed. Licensed for marketing in 1995, rFSH alfa derived from Chinese hamster ovary cells was the first available recombinant gonadotrophin preparation [73]. Further recombinant gonadotrophins have been developed and launched on the market since then, including rLH, rhCG, the combined product of recombinant follitropin alfa (rFSH alfa) and recombinant lutropin alfa in a 2:1 ratio and, more recently, long-acting rFSH alfa and the first human cell-line-derived rFSH delta [68–70, 74]. Despite a lack of evidence, it was assumed that recombinant products would demonstrate an improved batch-to-batch consistency compared with urinary-derived products. However, the new generation of highly purified urinary-derived gonadotrophin products, such as Menopur®, present a high standard of batch-to-batch consistency that is actually equivalent to that of recombinant products, such as Gonal-F® [77].

Following their introduction into the market, urinary-derived and recombinant gonadotrophins have been used in an equivalent manner for OS. A number of large clinical trials have investigated different gonadotrophin preparations (Table 2), which is leading to an improved understanding of the clinical differences between hCG and LH supplementation in OS.

**Table 2:** Overview of major clinical trials comparing HP-hMG versus rFSH and rFSH/rLH- versus rFSH-containing products in ART protocols

	Patients (N)	Treatment	Testing for	Primary outcomes
Menopur® studies				
**EISG** (EISG, Fertil Steril 2002 [10])	*N* = 781	**Menopur® versus Gonal-F®** 225 IU fixed dose for 5 days of either HP-hMG or rFSH alfa	Non-inferiority	**Efficacy:** OPR: 25% HP-hMG versus 22% rFSH alfa, *P* = 0.17 **Safety:** Adverse events probably and possibly related to the medication; 14.2% HP-hMG vs 13.0% rFSH alfa OHSS: 1.9% HP-hMG vs 1.2% rFSH alfa Miscarriage: 25.4% HP-hMG vs 27.6% rFSH alfa
**MERiT** (Andersen AN, et al. Hum Reprod 2006 [11])	*N* = 731	**Menopur® versus Gonal-F®** 225 IU fixed dose for 5 days of either HP-hMG or rFSH alfa, followed by individual adjustments according to the patient’s follicular response	Non-inferiority	**Efficacy:** OPR per started cycle: 27% HP-hMG vs 22% rFSH alfa (95% CI: 0.89 to 1.75, *P* = 0.204) **Safety:** Incidence of adverse events: 51% HP-hMG vs 49% rFSH alfa OHSS: 4% HP-hMG vs 3% rFSH alfa Early pregnancy loss: 26% HP-hMG vs 32% rFSH alfa
**Bosch E**, et al. (Hum Reprod 2008 [78])	*N* = 280	**Menopur® versus Gonal-F®** 225 IU fixed starting dose for 2 days of either HP-hMG or rFSH alfa, followed by individual adjustments according to serum oestradiol levels	Non- inferiority	**Efficacy:** OPR per randomised patient: 35.0% HP-hMG (95% CI: 27.1 to 43.5) vs 32.1% rFSH alfa (95% CI: 24.5 to 40.6) **Safety:** Cycles cancelled due to risk of OHSS: 6.4% HP-hMG vs 3.6% rFSH alfa, *P* = 0.27 Clinical OHSS: 1.64% HP-hMG vs 1.59% rFSH alfa Pregnancy loss: 0.82% HP-hMG vs 0.79% rFSH alfa
**MEGASET** (Devroey P, et al. Fertil Steril 2012 [12])	*N* = 749	**Menopur® versus Puregon®** 150 IU fixed dose for 5 days of either HP-hMG or rFSH beta, followed by dose adjustments if required of 75 IU increments, not more than every 4 days	Non-inferiority	**Efficacy:** OPR in the PP population: 30% HP-hMG versus 27% rFSH beta, 95% CI: 3.0% (− 3.8 to 9.8) **Safety:** Incidence of adverse events: 39% HP-hMG vs 37% rFSH beta OHSS: 3% in each treatment group Interventions associated with excessive response or to prevent early OHSS were higher in rFSH beta group (*P* = 0.025)
**MEGASET-HR** (Witz C, et al. Fertil Steril 2020 [79])	*N* = 620 Patients with serum AMH ≥5 ng/mL	**Menopur® versus Gonal-F®** 150 IU fixed start dose of either HP-hMG or rFSH alfa	Non-inferiority	**Efficacy:** OPR: 35.5% HP-hMG vs 30.7% rFSH alfa, 95% CI: 4.7% (−2.7 to 12.1) **Safety:** OHSS: 9.7% HP-hMG versus 21.4% rFSH alfa, 95% CI: − 11.7% (− 17.3 to − 6.1), *P* < 0.05 Incidence of treatment emergent adverse events: 57.7% HP-hMG vs 70.6% rFSH alfa Cumulative early pregnancy loss (fresh/frozen ET): 14.5% HP-hMG vs 25.5% rFSH alfa
**Taronger R**, et al. (Eur J Obstet Gynecol Reprod Biol 2018 [80])	*N* = 234 Poor ovarian responders	**Menopur® versus Elonva®** 150 μg single dose of CFA followed by 300 IU HP-hMG after Day 8 until criteria for triggering ovulation were met, versus 300 IU continuous daily dose of HP-hMG	Non-inferiority	**Efficacy:** OPR per started cycle: 20.2% HP-hMG vs 15.2 CFA, 95% CI: −5 (− 15.1 to 5.0), *P* = 0.33 **Safety:** Cancellation rate: 5.5% HP-hMG vs 3.6% CFA, *P* = 0.49
**Errázuriz J**, et al. (Front Endocrinol 2019 [81])	*N* = 917 Poor ovarian responders	**Menopur® versus Elonva®** 150 μg single dose of CFA followed by ≥300 IU HP-hMG after Day 8 until criteria for triggering ovulation were met, versus 7 x fixed daily doses of 300–450 IU HP-hMG	Non-inferiority	**Efficacy:** Cumulative LBR: 16.9% HP-hMG vs 11.8% CFA + HP-hMG; *P* = 0.03
Pergoveris® studies				
**PERSIST** (Behre, et al. RBM Online 2015 [82])	*N* = 202 Women aged 36–40 years	**Pergoveris® versus Gonal-F®** 300 IU fixed starting dose of rhFSH/rhLH from stimulation on Day 1 or rhFSH on stimulation on Days 1–5 followed by rhFSH + rhLH from stimulation Day 6. Dose adjustments were permitted from Day 6	Non-inferiority	**Efficacy:** Number of oocytes retrieved: 9.7 rhFSH/rhLH vs 10.9 rFSH alfa, 95% CI: –3.15 to 0.59 **Safety:** Adverse events reported: 31.1% rhFSH/rhLH vs 32.3% rFSH alfa Serious AEs: 2% rhFSH/rhLH vs 0% rFSH alfa OHSS: 3.9% rhFSH/rhLH vs 5.1% rFSH alfa
**ESPART** (Humaidan, et al. Hum Reprod 2017 [83])	*N* = 939 Poor ovarian responders	**Pergoveris® versus Gonal-F®** Fixed starting dose of 300 IU rhFSH + 150 IU rhLH (Pergoveris®) or 300 IU rFSH alfa (Gonal-F®). Dose adjustments were permitted from Day 4, at increments of 75 IU of rhFSH with a maximum daily dose of 450 IU (concomitant automatic adjustments of rhLH were made with a maximum daily dose of 325 IU)	Superiority	**Efficacy:** Number of oocytes retrieved: 3.3 rhFSH/rhLH vs 3.6 rFSH alfa, 95% CI: –0.24 (−0.74 to 0.27); *P* = 0.182 **Safety:** TEAEs: 19.9% rhFSH/rhLH vs 26.8% rFSH alfa Serious TEAEs: 1.7% rhFSH/rhLH vs 3.6% rFSH alfa, 95% CI: 0.46 (0.19 to 1.09) One incidence of OHSS in the rhFSH/rhLH group

*AE* adverse event, *ART* assisted reproductive technologies, *CFA* corifollitropin alfa, *CI* confidence interval, *CPR* clinical pregnancy rate, *ET* embryo transfer, *HP-hMG* highly purified human menopausal gonadotrophin, *LBR* live birth rate, *OHSS* ovarian hyperstimulation syndrome, *OPR* ongoing pregnancy rate, *PP* per protocol, *rFSH* recombinant follicle-stimulating hormone, *rhFSH* recombinant human follicle-stimulating hormone, *rhLH* recombinant human luteinising hormone, *rLH* recombinant luteinising hormone, *TEAE* treatment-emergent adverse event

## Clinical differences between hCG and LH

### End of stimulation endocrine environment and the impact on clinical outcomes

It is essential to consider all aspects when choosing the most appropriate gonadotrophin therapy for OS as different treatments could affect the end-of-stimulation endocrine environments, consequently impacting clinical outcomes and the overall goal of achieving a live birth. The MERiT trial (a prospective, randomised, controlled, multicentre study), primarily investigated the clinical outcome of 731 IVF patients treated with HP-hMG (Menopur®) versus rFSH alfa (Gonal-F®), while also collecting data on the endocrine profiles achieved in the patients following treatment [11, 84]. The trial was the subject of a *post-hoc* analysis carried out by Smitz et al., who found that, although there was no significant difference on stimulation Day 6 (*P* = 0.333), progesterone levels were significantly higher at the end of stimulation in the rFSH alfa group versus HP-hMG treatment (23% higher on the last day of stimulation, 3.4 ± 1.7 nmol/L vs 2.6 ± 1.3 nmol/L, *P* < 0.001; and 31% higher at oocyte retrieval, 36.3 ± 25 vs 24.5 ± 15.6, *P* < 0.001) [84]. The significance remained when adjusting for ovarian response (28% higher at the end of stimulation when adjusting for the number of follicles and 29% higher at oocyte retrieval when adjusting for the number of oocytes retrieved). In addition, a higher number of patients developed progesterone levels of > 4 nmol/L at the end of stimulation in the rFSH alfa group compared with the HP-hMG group (23% vs 11%, respectively), which was linked with reduced pregnancy rates [11, 84]. Oestradiol levels were found to be significantly higher by 20% in the rFSH alfa group on Day 6 of stimulation (1.1 ± 1.0 vs 1.0 ± 0.9, *P* = 0.004), 10% higher in the HP-hMG group by end of stimulation (7.2 ± 4.3 vs 6.6 ± 4.0, *P* = 0.031) and 16% higher at oocyte retrieval (3.9 ± 2.1 vs 3.4 ± 1.9, *P* = 0.001) [84]. Androstenedione levels were significantly increased in the HP-hMG versus rFSH alfa group at stimulation on Day 6, last stimulation day and after oocyte retrieval (Day 6: 6.0 ± 2.5 vs 5.5 ± 2.4, *P* = 0.002; last stimulation day: 11.9 ± 5.2 vs 9.5 ± 3.8, *P* < 0.001; oocyte retrieval: 13.6 ± 5.5 vs 10.8 ± 4.2, *P* < 0.001). When considering these results in combination with the outcomes of ongoing pregnancy rate (OPR) and live birth rate (LBR) from the primary study (OPR: 27% with HP-hMG group vs 22% with rFSH alfa, *P* = 0.204; LBR: 26% HP-hMG vs 22% rFSH alfa, *P* = 0.236), the data suggest that the differences in outcome observed between the HP-hMG and rFSH alfa groups may have been influenced by the different endocrine profiles induced by the respective treatments [11].

This hypothesis is further supported by the results from a retrospective study that analysed progesterone levels and OPR in more than 4000 OS cycles (IVF and intracytoplasmic sperm injection [ICSI] included in the analysis) and found that OPRs are inversely correlated with progesterone levels on the day of hCG administration. In addition, this study indicated that pregnancy rates are significantly higher in women with progesterone levels < 1.5 ng/mL than in those with progesterone levels > 1.5 ng/mL [85]. Furthermore, a meta-analysis of more than 60,000 fresh, frozen-thawed and donor/recipient IVF cycles, investigating the link between progesterone levels on the day of hCG administration and pregnancy outcomes, found that elevated progesterone levels are associated with a reduced probability of pregnancy in fresh cycles only (*P* < 0.05 for all thresholds defined as ≥ 0.8 ng/mL progesterone) [86]. Interestingly, elevated progesterone levels on the day of hCG administration were not associated with a reduced probability of pregnancy after frozen and donor/recipient IVF cycles, suggesting that a premature rise in progesterone levels during the follicular phase may act directly on the endometrium and impair its normal development. This may result in asynchrony between the embryo development and endometrium receptivity through advanced secretory transformations of the endometrium, thus shifting the implantation window so that it occurs earlier than normal in fresh cycles. A shifted implantation window could have subsequent detrimental effects on implantation and explain the poorer pregnancy outcomes that have been observed in clinical trials [87–93].

During assisted reproduction, ovaries are artificially stimulated in order to induce the development of multiple follicles for oocyte retrieval. In line with the ‘two-cell, two-gonadotrophin theory’, administration of supraphysiological levels of FSH stimulates the development of multiple follicles, consequently increasing the production of progesterone [94]. Treatment supplementation with LH or hCG during the stimulation is thought to instead drive the conversion of pregnenolone to androgens (via the Delta 5 pathway), which are further converted by FSH into oestrogens, thereby limiting the conversion of pregnenolone to progesterone [95, 96]. As a result, less progesterone is available to enter the bloodstream [88]. Data from a recent clinical trial that studied the follicular steroidogenesis pathways and progesterone levels in oocyte donors treated with either rFSH alfa or HP-hMG for OS support the importance of attaining a balance of FSH and LH bioactivity when stimulating using gonadotrophins [97]. Indeed, there was a significant increase in serum progesterone following rFSH alfa stimulation compared with HP-hMG stimulation both on the day of trigger (0.68 ± 0.50 ng/mL vs 0.46 ± 0.27 ng/mL, respectively; *P* = 0.010), and on stimulation Day 8. Consistently, treatment with HP-hMG was associated with a significant increase in androstenedione compared with rFSH alfa (3.0 ± 1.4 vs 2.4 ± 1.1, respectively; *P* = 0.015) on both stimulation days, and a significantly higher pregnenolone:progesterone ratio on the day of trigger (*P* = 0.019). Although the ovarian response remained comparable between the two groups (17.5 ± 7.9 oocytes retrieved in the rFSH alfa group vs 16.5 ± 7.5 in the HP-hMG group, *P* = 0.49), these results indicate that different follicular steroidogenesis pathways are at play with rFSH alfa versus HP-hMG OS.

Several reviews have recently discussed the controversy surrounding the origin of premature progesterone elevation in OS based on evidence to date [90, 93, 98]. Initially, it was hypothesised that the LH bioactivity of hMG preparations may cause elevations in circulating progesterone in the context of premature LH surges. Contrary to expectations, studies comparing circulating progesterone levels following the administration of FSH or hMG revealed that FSH was associated with a similar or an increased elevation in progesterone compared with hMG (progesterone levels increased from 0.37 ± 0.12 ng/mL before FSH administration to 0.86 ± 0.12 ng/mL approximately 15 h post-administration, *P* < 0.01 [93, 99]). These data suggest that FSH stimulation alone could be associated with elevated circulatory progesterone levels at the end of stimulation, but did not distinguish between the individual influences of hCG and LH. A study by Sebag-Peyrelevade et al. (2015) analysed whether supplementation of rFSH alfa treatment with rLH could lead to comparable progesterone levels on the day of hCG triggering compared with HP-hMG (Menopur®) treatment alone. The study, conducted on pituitary-desensitised IVF patients, revealed that rFSH alfa supplementation with rLH was not sufficient to diminish the circulating progesterone levels to the same level as those receiving HP-hMG (median of 0.63 ng/mL for HP-hMG vs 0.91 ng/mL for rLH/rFSH alfa median, *P* < 0.0001). This relationship remained significant even when adjusted for the number of growing follicles to control for the extent of ovarian response (0.055 ng/mL/growing follicle vs 0.077 ng/mL/growing follicle) [100]. A lack of effect was also reported following administration of a higher rLH dose in the rFSH alfa/rLH group: it was, therefore, suggested that the lower progesterone levels induced with HP-hMG treatment may be attributable to the hCG content (not LH) [100].

Interestingly, embryo quality may be equally as important in determining pregnancy outcomes as the endometrial receptivity induced by elevated progesterone levels. It has been suggested that patients with a good ovarian response produce oocytes/embryos of increased quality, and so may be better equipped to overcome the challenges presented by an impaired endometrial receptivity brought about by high progesterone levels. In contrast, patients with a poor ovarian response are more likely to produce oocytes of lower quality, which are less likely to be able to compensate for the impaired endometrial receptivity, thus resulting in a lower OPR [93, 98]. Data from multiple clinical trials have indicated that a progesterone level > 0.9 ng/mL on the day of hCG administration is associated with lower pregnancy rates in patients with a weak ovarian response. Therefore, it has been speculated that previous trials not reporting a link between premature elevated progesterone levels and reduced pregnancy outcomes may have unknowingly included in their analyses a majority of normal or high ovarian responders, with resulting good-quality embryos being capable of compensating for impaired endometrial receptivity [93]. Taken together, these findings indicate that OS could potentially be adapted to prevent progesterone elevation by individualisation according to a patient’s predicted ovarian response and embryo quality [101].

### Number of oocytes retrieved and proportion of top-quality embryos achieved with different gonadotrophin preparations

Although live birth is the most clinically meaningful goal of ART, the number of oocytes retrieved following OS is frequently used as a surrogate measure of clinical success [102]. Data from several studies indicate that rFSH alfa and beta trigger an increased ovarian response, in terms of number of oocytes retrieved, compared with HP-hMG. In a study by Bosch et al. (2008), in which 280 patients undergoing IVF/ICSI were randomised to receive HP-hMG (Menopur®) or rFSH alfa (Gonal-F®) in GnRH antagonist protocols, the numbers of cumulus–oocyte complexes (COCs) retrieved and metaphase II (MII) oocytes obtained (ICSI cycles) were significantly higher with rFSH alfa than HP-hMG (14.4 ± 8.1 vs 11.3 ± 6.0, *P* = 0.001 and 9.7 ± 6.0 vs 7.8 ± 4.0, *P* = 0.004, respectively) [78]. The MERiT [11] and MEGASET [12] studies also reported higher numbers of oocytes retrieved in the rFSH alfa and beta groups (respectively) than in the HP-hMG group (11.8 ± 5.7 vs 10.0 ± 5.4, *P* < 0.001 and 10.7 ± 5.8 vs 9.1 ± 5.2, *P* < 0.001, respectively). These results were further replicated in the recently concluded MEGASET-high responder (MEGASET-HR) trial, in which 620 women predicted to be high responders were randomised to receive either HP-hMG (Menopur®) or rFSH alfa (Gonal-F(R)) in a GnRH antagonist cycle [13, 14, 79]. As in previous studies, the number of oocytes retrieved was higher in the rFSH alfa arm than in the HP-hMG arm (22 ± 11.54 vs 15.1 ± 10.12) [14, 79]. Interestingly, the PERSIST trial comparing a 2:1 formulation of rFSH alfa plus rLH (Pergoveris®) administered from Day 1 versus rFSH alfa (Gonal-F®) administered during Days 1–5 and supplemented with rLH from Day 6 of the stimulation cycle did not report a significant difference in terms of oocytes retrieved (9.7 vs 10.9, 95% confidence interval [CI]: − 3.15 to 0.59), therefore failing to meet its primary endpoint and yielding inconclusive evidence on the influence of rLH on oocyte yield [82].

To understand whether differences in the source of LH activity affect ovarian stimulation characteristics (number of oocytes retrieved and percentage of mature oocytes) and IVF outcome, a critical appraisal of studies comparing hMG and rFSH alfa/beta + rLH was performed [103]. Of the 11 studies included, most were observational, with only two randomised controlled trials (RCTs) evaluated. Moreover, only one of the RCTs included compared hMG and rLH from day 1 of ovarian stimulation [104]. This RCT, performed on 111 patients, found that hMG was associated with a longer length of treatment but, surprisingly, a lower total amount of FSH administered compared with the rFSH alfa + rLH group. Notably, this study was performed in Italy at a time when only 3 oocytes were permitted to be inseminated by law, an additional reason to exert caution when interpreting these results. The author found that there was insufficient evidence to form any firm conclusions on whether the source of LH activity affects ovarian stimulation characteristics and concluded that further RCTs on this subject are needed [103].

Although studies consistently show that rFSH alfa and beta stimulation is associated with the retrieval of a higher number of oocytes than HP-hMG, the data also indicate that rFSH alfa/beta and HP-hMG stimulation may influence the quality of the oocytes retrieved, and this, in turn, could impact clinical outcomes. In the MERiT study, a higher proportion of retrieved oocytes developed into high-quality embryos in the HP-hMG group than in the rFSH alfa group (11.3 ± 16.1% versus 9.0 ± 13.0%, *P* = 0.044) [11, 105]. The MEGASET trial then reported how, despite the higher number of oocytes retrieved in the rFSH beta group, the number and quality of blastocysts on Day 5 was comparable between the two treatment arms [12]. Again, in the MEGASET-HR study, the difference in the number of oocytes retrieved, as calculated by the Hodges-Lehmann estimate, was seven fewer with HP-hMG compared with rFSH alfa, which narrowed to zero for excellent quality blastocysts (numerical difference of 0.9). MEGASET-HR also reported cumulative higher rates of early pregnancy loss in the rFSH alfa treatment arm, suggesting poor oocyte quality in comparison with the HP-hMG treatment arm [79]. Taken together, these data suggest that treatment with rFSH alfa/beta is likely to result in a higher oocyte yield compared with HP-hMG, but that the quality of oocytes produced with HP-hMG is proportionally higher than that produced with rFSH alfa/beta. Oocyte quality-related issues may explain why the incremental oocyte yield with rFSH alfa/beta does not translate into a similarly augmented abundance in numbers of good-quality blastocysts between the two groups. Furthermore, these studies also highlight why oocyte yield is not a clinically meaningful endpoint to evaluate ART efficiency.

### The impact of improved embryo quality on OPR and LBR

#### Normal responders

The higher proportion of top-quality embryos achieved with HP-hMG is suggested to be the driver for an improved implantation rate, OPR and LBR compared with rFSH alfa/beta in both IVF and ICSI treatment cycles [12, 105]. Results from the MERiT study in normal responders indicated that top-quality embryos obtained from HP-hMG-treated patients are associated with a numerically higher OPR and LBR compared with top-quality embryos from rFSH alfa-treated patients (OPR: 27% HP-hMG vs 22% rFSH alfa, odds ratio [OR]: 1.25, 95% CI: 0.89 to 1.75, *P* = 0.204; LBR: 26% HP-hMG vs 22% rFSH alfa, *P* = 0.236) [11]. Interestingly, the concentration of hCG during the stimulation was significantly associated with LBR [106, 107]. The embryo quality in this study was assessed both by local embryologists via live microscopy (local assessment), as well as by a panel of three central embryologists via photographic evidence (central assessment). The significant difference in the proportion of top-quality embryos retrieved (11.3% in the HP-hMG treatment arm vs 9% in the rFSH alfa treatment arm, *P* = 0.044) was observed only in the local assessment [11]. Although non-significant, a similar increase in the number of top-quality embryos in the HP-hMG group was identified by central assessment [105].

Subsequent investigations in normal responders in the MEGASET study provided data showing that, despite a significantly reduced number of oocytes retrieved in the HP-hMG group (9.1 ± 5.2 in the HP-hMG group vs 10.7 ± 5.8 in the rFSH beta group, *P* < 0.001) and a similar number of top-quality embryos between groups (31 ± 30% in the HP-hMG group vs 31 ± 28% in the rFSH beta group, *P* = 0.546), there was a non-significant trend towards an increased OPR with HP-hMG relative to rFSH beta (30% vs 27, 95% CI: − 3.8 to 9.8). Cumulative LBR, recorded for patients who underwent a single stimulation cycle with a single fresh or frozen blastocyst transfer within a year of treatment initiation, were similar in both groups, with a positive trend in favour of HP-hMG (40% HP-hMG vs 38% rFSH beta). These results met the primary endpoint of non-inferiority and indicated that HP-hMG is as effective as rFSH beta [12].

#### Poor responders

The data for embryo quality, OPR and LBR have been similarly improved with HP-hMG in poor ovarian responders. Due to a reduced ovarian reserve, it can be particularly difficult to achieve a satisfactory ovarian response, measured through the number of oocytes retrieved, which can result in poor ART outcomes in this patient population. Some investigators have focused their attention on the use of long-acting products in order to increase the level of stimulation that the ovaries are subjected to, in the hope of producing a higher oocyte yield in this patient population. A recent prospective, randomised study investigated the use of a single daily dose of long-acting corifollitropin alfa (CFA) (Elonva®) plus HP-hMG from cycle Day 8 (HP-hMG was only administered after Day 8 if required until the criteria for ovulation triggering were achieved), compared with a continuous daily dose of HP-hMG (Menopur®) for OS in 234 patients (< 40 years of age) who were at risk of poor ovarian response [80]. HP-hMG was associated with a significantly higher number of MII oocytes retrieved (3.8 vs 3.1, *P* = 0.04), a higher number of embryos on the day of transfer (2.2 vs 1.7, *P* = 0.05) and an increased number of cycles with vitrified embryos (18% vs 9%, *P* = 0.05). Although this did not translate into a significant difference in the number of top-quality embryos transferred (1.2 HP-hMG group vs 1.1 CFA, *P* = 0.60) or the primary endpoint of OPR (20.1 vs 15.2, 95% CI: − 15.1 to − 5.0), there was a trend towards increased OPR, LBR and cumulative LBR in patients who received HP-hMG-only treatment, which the authors concluded may be clinically relevant. When comparing this to the retrospective analysis, which included 917 patients characterised as poor responders (according to the Bologna criteria for poor ovarian response) and treated with either a fixed daily dose of 300–450 IU of HP-hMG (Menopur®) or a single dose of CFA (Elonva®), followed by daily injections of HP-hMG from Day 8 of stimulation until the day of trigger, similar results were observed in terms of a reduced number of top-quality embryos achieved with CFA [81]. The number of COCs and MII oocytes were significantly higher for the HP-hMG treatment group (*P* < 0.001 and *P* = 0.004, respectively), and the number of patients with at least one top-quality embryo for transfer was higher in the HP-hMG group on both embryo transfer Days 3 and 5 (Day 3: 595 HP-hMG vs 444 CFA + HP-hMG, *P* = 0.088; Day 5: 32 HP-hMG vs 24 CFA + HP-hMG, *P* = 0.724). Investigators did not report significant differences in the LBR between patients treated; however, the secondary outcomes of biochemical pregnancy, clinical pregnancy and OPR were significantly higher for the HP-hMG treatment group compared with the CFA + HP-hMG group (biochemical pregnancy: 26% vs 18%, *P* = 0.007; clinical pregnancy: 23% vs 16%, *P* = 0.006; and OPR 17% vs 11%, *P* = 0.008). Additionally, HP-hMG achieved a non-significant, numerically higher LBR (14% vs 10%, *P* = 0.09) [81]. Overall, these data indicate that HP-hMG-only protocols may be associated with increased numbers of high-quality oocytes in poor responders. This could be particularly beneficial in this patient population as expected oocyte yield in poor responders is low.

Another treatment that has been investigated in poor responders is a 2:1 formulation of rFSH alfa plus rLH (Pergoveris®). Despite its therapeutic indication solely for ovarian induction in patients with hypogonadotropic hypogonadism (severe LH and FSH deficiency), clinicians continue to use this product for OS in poor responders due to its rLH content. Importantly, when investigated during the ESPART trial [83], a large RCT undertaken to determine if there is a difference in the efficacy and safety of rFSH alfa/rLH (Pergoveris®) versus rFSH alfa monotherapy (Gonal-F®) when administered for OS in poor responders (classified according to a modified version of the Bologna criteria), the study failed to show superiority of rFSH alfa/rLH versus rFSH alfa monotherapy in regard to the primary endpoint of number of oocytes retrieved (3.3 rFSH alfa/rLH vs 3.6 rFSH alfa; adjusted *P*-value of 0.182). Furthermore, the secondary endpoints of clinical and OPR, as well as LBR, were similar between the two groups (clinical pregnancy: 14.1% rFSH alfa/rLH vs 16.8% rFSH alfa, *P* = 0.320; OPR: 11% rFSH alfa/rLH vs 12.4% rFSH alfa, *P* = 0.599; LBR: 10.6% rFSH alfa/rLH vs 11.7% rFSH alfa, *P* = 0.663). The rate of biochemical pregnancy was significantly higher in the rFSH alfa monotherapy group (17.3% rFSH alfa/rLH vs 23.9% rFSH alfa, *P* = 0.020); however, this significance did not carry through to ongoing pregnancy. Considering these data alongside the trials comparing HP-hMG with CFA in poor responders, the results suggest that the positive outcomes in terms of oocyte yield associated with HP-hMG-only protocols may be attributable to the hCG content (not LH).

#### High responders

Retrospective analyses of the data from MERiT and MEGASET trials investigated the ovarian response and clinical outcomes in potential high responders [106, 108]. Women were grouped according to their serum anti-Müllerian hormone (AMH) levels, and those with AMH values in the highest quartile (> 5.2 ng/ml) were identified as potential high responders. The analysis revealed that HP-hMG was associated with a favourable safety profile, with HP-hMG treatment resulting in a lower incidence of high response (≥15 oocytes retrieved) compared with rFSH alfa/beta (MERiT: 33% in the HP-hMG group vs 51% in the rFSH alfa group, *P* = 0.025; MEGASET: 31% in the HP-hMG group vs 49% in the rFSH beta group, *P* = 0.015) [106]. In the MEGASET trial, HP-hMG also showed a trend towards a lower incidence of early ovarian hyperstimulation syndrome (OHSS) and/or safety interventions due to excessive ovarian response compared with rFSH beta (*P* = 0.025) [108]. In addition, a shift towards an improved LBR was observed with HP-hMG compared with rFSH alfa/beta (combined studies: 34% in the HP-hMG group vs 22% in the rFSH alfa/beta group, *P* = 0.012), despite the fact that a lower oocyte yield was achieved (MERiT: 12 in the HP-hMG group vs 15 in the rFSH alfa group, *P* = 0.007; MEGASET: 12 in the HP-hMG group vs 14 in the rFSH beta group, *P* = 0.033) [106, 108]. These data suggest that HP-hMG is associated with a reduced risk of high response rates compared with rFSH alfa/beta, which may influence clinical outcomes in predicted high responders. Furthermore, these data indicate that a greater oocyte yield does not necessarily translate into improved pregnancy outcomes in this patient population. To investigate these findings further, a prospective trial was conducted (MEGASET-HR) specifically to analyse the effects of HP-hMG treatment in a predicted high responder population [13, 14, 79].

The recent MEGASET-HR trial comparing HP-hMG and rFSH alfa in predicted high responders found that HP-hMG was associated with a trend towards increased OPR/cycle start relative to rFSH alfa (OPR 35.5% vs 30.7, 95% CI: − 2.7 to 12.1), despite a reduced oocyte yield (HP-hMG 15.1 vs rFSH alfa 22.2). These results may at least partly be explained by the fact that the difference in the mean number of oocytes per patient narrowed with each subsequent oocyte developmental stage analysed. Indeed, at the stage of excellent quality blastocysts, the mean difference was 0% (95% CI: − 1.0 to 0.0) [79]. A possible theory explaining this discrepancy between treatments could be that HP-hMG, more than rFSH alfa, facilitates selection of high-quality embryos in the ovary, prior to oocyte retrieval. Cumulative live birth rate per cycle start was comparable between HP-hMG and rFSH alfa (HP-hMG 50.6% vs rFSH alfa 51.5%), despite a higher total number of embryos transferred in the rFSH group (HP-hMG 308 vs rFSH alfa 373). The lack of clinical efficacy conferred by a higher number of blastocysts in the rFSH alfa group could partly be explained by a higher live birth rate per transfer and lower early pregnancy loss in HP-hMG-treated subjects in both fresh and frozen transfer cycles. The MEGASET-HR trial also reported that HP-hMG was associated with a significantly reduced rate of OHSS compared with rFSH alfa treatment (9.7% HP-hMG vs 21.4% rFSH alfa, 95% CI: − 17.3 to − 6.1). These findings support the non-inferiority of HP-hMG versus rFSH alfa with respect to efficacy in patients predicted to be high responders undergoing ICSI, and suggest that the increased ovarian response and number of retrieved oocytes seen with rFSH alfa treatment in this patient group may translate into diminished pregnancy outcomes in terms of OPR, cumulative pregnancy loss and risk of OHSS [13, 14, 79].

#### Meta-analyses

In addition to the aforementioned clinical trials, a number of meta-analyses have been conducted to examine the published data for pregnancy outcomes with different gonadotrophin products. Two separate meta-analyses have reported similar trends for an increased LBR and clinical pregnancy rate (CPR) with hMG: Coomarasamy et al. (2007) analysed seven studies, with a total of 2159 women, comparing hMG with rFSH alfa/beta treatment as part of IVF/ICSI treatment within a GnRH agonist protocol [109], whereas Al-Inany and colleagues (2008) pooled data from 12 studies comparing the efficacy and safety of hMG and rFSH alfa/beta in 3575 women [110]. Both concluded that LBR (relative risk [RR] = 1.18, 95% CI: 1.02 to 1.38; OR: 1.20, 95% CI: 1.01 to 1.42) and CPR (RR = 1.17, 95% CI: 1.03 to 1.34; OR: 1.22, 95% CI: 1.03 to 1.43) were significantly increased with hMG treatment versus rFSH alfa/beta alone.

A separate, large meta-analysis investigated pregnancy outcomes with rFSH alfa/beta treatment versus urinary-derived gonadotrophins (hMG, purified FSH or HP-FSH), including 42 trials and more than 9000 couples in the analysis. The investigators concluded that there were no significant differences in the LBR or OHSS rates with rFSH alfa/beta versus all other gonadotrophin treatments combined [111], although when individual urinary gonadotrophins were considered separately there were significantly fewer live births in the rFSH alfa/beta group when compared with hMG alone (OR: 0.84, 95% CI: 0.72 to 0.99; *N* = 3197), which is consistent with the findings of the previous two meta-analyses.

A recently published systematic review and meta-analysis investigated whether recombinant gonadotrophins differ from HP-hMG in terms of the total amount required to achieve a live birth. A total of seven RCTs with 3220 women were included, all of which directly compared rFSH alfa/beta with HP-hMG for OS. Although pooled analyses of the seven studies did not provide evidence of a difference in the amount of gonadotrophins used per woman that started an IVF/ICSI cycle (− 37 IU, 95% CI: − 115 to 41; I^2^ = 68%), it did reveal some significant differences between gonadotrophins in terms of outcomes. The difference in the mean gonadotrophin amount per extra live birth was 789 IU (95% CI: − 9.5 to 1570) for rFSH alfa/beta versus HP-hMG, and treatment with HP-hMG was associated with a significantly higher LBR compared with rFSH (RR: 0.88, 95% CI: 0.78 to 0.99, *P* = 0.03). There was insufficient evidence to detect a difference in cumulative LBR between rFSH alfa/beta and HP-hMG (RR: 0.91, 95% CI: 0.80 to 1.04, *P* = 0.17), which may be because only three of the seven studies reported this endpoint and there were differences in cryopreservation techniques between studies.

Taken together, the clinical studies and meta-analyses discussed support of the non-inferiority of HP-hMG compared with rFSH alfa/beta regarding efficacy and safety. Furthermore, the growing body of evidence suggests that HP-hMG leads to incremental benefits in key pregnancy outcomes compared with rFSH alfa/beta, which translates to a higher LBR, as seen in meta-analyses. These benefits may be at least partly due to the improved oocyte quality associated with HP-hMG treatment and a better synchronisation of the endometrium, a finding reported across several clinical trials.

## Conclusions

Although hCG and LH were previously believed to have overlapping roles in the reproductive system, the biological and clinical data discussed in this review highlight emerging evidence suggesting that they have distinct functional differences throughout the menstrual cycle and normal physiology, which can ultimately impact responses and outcomes in ART. However, because LH and hCG bind to a common receptor, there is an ongoing scientific debate over the optimal use of products that derive their LH bioactivity from either hCG or from LH.

Some clinicians believe that there is an established need for LH bioactivity in OS, which could be met through supplementation with rLH products. However, evidence to the contrary was provided by the PERSIST and ESPART trials, which indicated that rFSH alfa/rLH treatment is neither equivalent nor superior to rFSH alfa monotherapy for the number of oocytes retrieved, OPR or LBR, with the latter two outcomes displaying a negative trend instead. Data further suggest that poor responders may benefit from HP-hMG treatment, particularly to achieve an adequate oocyte yield. Oocyte yield is an important issue as it is thought to contribute to treatment efficacy.

Several studies, including EISG [10], MERiT [11], MEGASET [12] and MEGASET-HR [79], have consistently provided evidence in favour of LH bioactivity derived from hCG in OS [78, 80, 81]. These studies suggest that, although associated with a lower oocyte yield, HP-hMG treatment may result in a higher proportion of good-quality embryos and/or may increase endometrial receptivity compared with rFSH alfa/beta. Randomised controlled trials have indicated a consistent trend towards an improved OPR and LBR following fresh embryo transfer and HP-hMG treatment. These effects may be partly related to the fact that HP-hMG is associated with a lower risk of premature progesterone rises during OS [100]. An alternative explanation could be related to the higher proportion of good-quality embryos associated with HP-hMG: could the evidence be suggesting that HP-hMG facilitates the selection of good-quality oocytes within the body, while the selection takes place in the laboratory when patients are treated with rFSH alfa/beta?

Overall, the studies reviewed here indicate that hCG supplementation and the use of hCG-containing products are efficacious and ultimately lead to an improved LBR in normal and poor responders. In addition, there is evidence that hCG-containing products result in at least comparable, if not reduced, rates of OHSS and/or safety interventions due to excessive ovarian response to gonadotrophin preparations in high responders. Together, these data provide robust evidence that hCG-containing products are a valuable tool for OS in the field of ART.

## Data Availability

Not applicable.

## Abbreviations

AC: Adenylyl cyclase enzyme;
AE: Adverse event
AKT: Protein kinase B
ART: Assisted reproductive technologies
ATP: Adenosine tri-phosphate
cAMP: cyclic adenosine monophosphate
CFA: Corifollitropin alfa
CG: Chorionic gonadotrophin
CI: Confidence interval
COC: Cumulus–oocyte complex
COOH: Carboxylic acid
CREB: cAMP response element-binding protein
CTP: Carboxy-terminal peptide
EGF: Epidermal growth factor
ERK1/2: Extracellular signal-regulated kinase ½
FSH: Follicle-stimulating hormone
GnRH: Gonadotrophin-releasing hormone
hCG: Human chorionic gonadotrophin
hLH: Human luteinising hormone
hMG: Human menopausal gonadotrophin
HP-FSH: Highly purified FSH
HP-hMG: Highly purified human menopausal gonadotrophin
ICSI: Intracytoplasmic sperm injection
IVF: In vitro fertilisation
LBR: Live birth rate
LH: Luteinising hormone
LH/CGR: Luteinising hormone/choriogonadotrophin receptor
OHSS: Ovarian hyperstimulation syndrome
OPR: Ongoing pregnancy rate
OR: Odds ratio
OS: Ovarian stimulation
PDE: Phosphodiesterase enzyme
PKA: Protein kinase A
Pl3K: Phosphoinositide 3-kinases
PP: Per protocol
RCT: Randomised controlled trial
rFSH: Recombinant follicle-stimulating hormone;
rhCG: Recombinant hCG
rhFSH: Recombinant human follicle-stimulating hormone
rhLH: Recombinant human luteinising hormone
rLH: Recombinant LH
TEAE: Treatment-emergent adverse event
